# Identification of individual subjects based on neuroanatomical measures obtained 7 years earlier

**DOI:** 10.1111/ejn.15770

**Published:** 2022-08-01

**Authors:** Lutz Jäncke, Seyed A. Valizadeh

**Affiliations:** ^1^ Division Neuropsychology, Department of Psychology University of Zurich Zurich Switzerland; ^2^ University Research Priority Program “Dynamics of Healthy Aging” University Zurich Zurich Switzerland; ^3^ Present address: ETH Zürich, Department of Humanities, Social and Political Sciences Zurich Switzerland

**Keywords:** FreeSurfer, linear discriminant analysis, MRI, random forest, rule‐based identification approach, subject authentication, subject identification

## Abstract

We analysed a dataset comprising 118 subjects who were scanned three times (at baseline, 1‐year follow‐up, and 7‐year follow‐up) using structural magnetic resonance imaging (MRI) over the course of 7 years. We aimed to examine whether it is possible to identify individual subjects based on a restricted number of neuroanatomical features measured 7 years previously. We used FreeSurfer to compute 15 standard brain measures (total intracranial volume [ICV], total cortical thickness [CT], total cortical surface area [CA], cortical grey matter [CoGM], cerebral white matter [CeWM], cerebellar cortex [CBGM], cerebellar white matter [CBWM], subcortical volumes [thalamus, putamen, pallidum, caudatus, hippocampus, amygdala and accumbens] and brain stem volume). We used linear discriminant analysis (LDA), random forest machine learning (RF) and a newly developed rule‐based identification approach (RBIA) for the identification process. Using RBIA, different sets of neuroanatomical features (ranging from 2 to 14) obtained at baseline were combined by if–then rules and compared to the same set of neuroanatomical features derived from the 7‐year follow‐up measurement. We achieved excellent identification results with LDA, while the identification results for RF were very good but not perfect. The RBIA produced the best results, achieving perfect participant identification for some four‐feature sets. The identification results improved substantially when using larger feature sets, with 14 neuroanatomical features providing perfect identification. Thus, this study shows again that the human brain is highly individual in terms of neuroanatomical features. These results are discussed in the context of the current literature on brain plasticity and the scientific attempts to develop brain‐fingerprinting techniques.

## Graphical abstract

We analysed an magnetic resonance imaging (MRI) dataset comprising 118 subjects who were scanned three times over a period of 7 years using 15 standard brain measures. Applying linear discriminant analysis, random forest machine learning, and a newly developed rule‐based identification approach (RBIA) revealed excellent identification of individual subjects. The RBIA produced the best results, achieving perfect participant identification for some four‐feature sets and perfect identification with 14 neuroanatomical features. Thus, this study shows again that the human brain is highly individual in terms of neuroanatomical features.

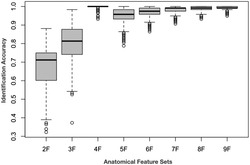

**Abbreviations**
ACC: accuracy
CA: total cortical surface area
CBGM: cerebellar cortex
CBWM: cerebellar white matter
CeWM: cerebral white matter
CoGM: cortical grey matter (CoGM)
CT: total cortical thickness
F1‐score: harmonic mean of sensitivity and precision (the proportion of truly classified subjects over subjects' number in the class, also called positive predictive value)
FOV: field of view
ICV: Intracranial volume
IQ: psychometric intelligence
LDA: linear discriminant analysis
LHAB: Longitudinal Healthy Aging Brain
MMSE: Mini‐Mental State Examination
MRI: magnetic resonance imaging
RBIA: rule‐based identification approach
RF: random forest
ROI: region of interest
Sen: Sensitivity
SF‐12: 12‐Item Short Form Survey
Spec: specificity

## 1 INTRODUCTION

In a previous study, our group showed that it is possible to identify individual subjects based on neuroanatomical features obtained from regular structural magnetic resonance imaging (MRI) scans and subsequent analysis with the popular FreeSurfer tool (Valizadeh et al., 2018). Identification rates have been very good, even when using only a few neuroanatomical features (11 brain measures comprising total brain volume, cerebellar grey and white matter, basal ganglia volumes and brain stem volume). When using a large set of brain regions, the subject identification rates became nearly perfect. The precision of subject identification using easy‐to‐obtain neuroanatomical measures resembled the identification results reported by others using more sophisticated neuroanatomical measures (Wachinger et al., 2015, 2017). These results are taken as evidence that the human brain is, to a large part, highly individual.

The search for individual markers based on neuroscientific methods and data has become very popular in recent years. Recent research in this area has shown that individual subjects can be differentiated and identified based on neural fingerprints derived from structural MRI (Wachinger et al., 2015, 2017; Valizadeh et al., 2018), functional MRI (Miranda‐Dominguez et al., 2014; Finn et al., 2015; Amico & Goñi, 2018; Bari et al., 2019), electroencephalography (EEG) (La Rocca et al., 2014; Fraschini et al., 2015; Kong et al., 2019; Valizadeh et al., 2019) or functional near‐infrared spectroscopy (fNIRS) (de Souza Rodrigues et al., 2019). Currently, it has also been suggested that such neural fingerprints might be related to individual differences in intelligence and fluid cognitive abilities, such as working memory and attention (Greene et al., 2018; Rosenberg et al., 2020; Yamashita et al., 2018; Yoo et al., 2018). It could also be possible that individual fingerprints accumulate to group fingerprints discriminating clinical populations. This brain‐fingerprint research emerges at the same time at which large, openly available datasets are available. However, the big‐data neuroscience approach often neglects the individuality, singularity, and variability of human beings. Thus, to understand this individual variability, it is necessary to delineate the individual characteristics of the human brain.

In our previous study, we used a dataset of 193 older adult subjects, from whom MRI data were obtained annually over 3 years (Valizadeh et al., 2018). Of the three scans obtained per subject, two were randomly selected for training the machine learning algorithms, while the third scan was used for testing. Scans were selected randomly to ensure no bias regarding identification. For many subjects of this study, 4‐ and 7‐year follow‐up scans have been acquired and recent analyses of these data indicate substantial neuroanatomical changes (Hotz et al., 2021; Jäncke et al., 2020; Sele et al., 2020, 2021). Thus, we are interested to examine the precision of subject identification when the training of the classification algorithms is based on neuroanatomical data obtained during the first two measurement occasions and the testing (e.g., subject identification) is based on data obtained 7 years after the baseline scan when substantial neuroanatomical changes have taken place.

For our project, we used two identification tools that have been shown to produce robust and valid results: the linear discriminant analysis (LDA) and the random forest (RF) machine learning algorithms. A major advantage of both algorithms is that they allow the calculation of the relative importance of each variable (in this case, neuroanatomical measures) in contributing to subject identification. Other popular and useful machine learning algorithms, such as weighted k‐nearest neighbour (WKNN) and nearest neighbour (NN), do not permit this calculation. In addition, we developed a rule‐based identification approach (RBIA). This approach is based on simple, logical if‐then rules, which combine the different anatomical features obtained at baseline to identify individual subjects 7 years later.

As neuroanatomical measures, we used easy‐to‐obtain measures based on the FreeSurfer tool for all three identification tools (LDA, RF and RBIA). To avoid overfitting as with LDA, we restricted our analysis to a relatively small set of features (comprising 15 measures, which are in principle similar to those we used previously). These neuroanatomical measures include total cortical thickness and surface area since it has been shown that thickness and surface area are two different neuroanatomical traits associated with different neurophysiological and psychological issues (Hogstrom et al., 2013; Rakic, 1995, 2000). Furthermore, we included volume measures for the cortex, cerebellum and subcortical areas. The subcortical areas comprise brain structures that we have shown degenerate the most within 7 years (e.g., hippocampus and accumbens) (Sele et al., 2020).

With this study, we aim to answer the following questions: (1) Is it possible to identify individual subjects based on a combination of anatomical measures when the training of the classification algorithms is based on anatomical data that is 7 years old? (2) Which combination of anatomical measures is most informative in identifying individual subjects? (3) Do the different classification techniques (linear discriminant analysis: LDA and random forest: RF) substantially differ in terms of their subject identification accuracy? (4) How accurate are relatively simple if‐then rules based on anatomical measures obtained at baseline and 1‐year follow‐up for identifying individual subjects 7 years later?

## 2 METHOD

### 2.1 Subjects

Structural MRI data were taken from the Longitudinal Healthy Aging Brain Database Project (LHAB; Switzerland)—an ongoing project conducted at the University of Zürich. In this project, healthy older subjects were scanned and tested with neuropsychological tests at five measurement occasions (baseline, 1‐year‐follow‐up, 2‐year follow‐up, 4‐year‐follow‐up and 7‐year‐follow‐up). For 24 subjects MRI data were additionally collected at a 3‐year follow‐up. The LHAB dataset included 232 participants at baseline, of which 231 had MRI data (age at baseline: M = 70.8, range = 64–87; females: 113). A detailed description of the sample characteristic has been provided in previous papers of our group (Oschwald et al., 2019, Oschwald et al., 2021; e.g., Jäncke et al., 2020; Malagurski et al., 2020). Thus, we only briefly reiterate the procedure and basic data here.

At each measurement occasion, participants underwent extensive brain imaging and completed a battery of neuropsychological and cognitive tests. Inclusion criteria for study participation at baseline were age ≥64, right‐handedness, fluent German language proficiency, a score of ≥26 on the Mini‐Mental State Examination (MMSE; Folstein et al., 1975), no self‐reported neurological disease of the central nervous system and no contraindications to MRI. Participation was voluntary and all participants gave written informed consent in accordance with the declaration of Helsinki. The self‐reported physical and mental health of the sample at baseline, as measured by the 12‐Item Short Form Survey (SF‐12, Ware et al., 1996), indicated above‐average health compared to a normal population. The mean IQ of the sample was 120.6 (*SD* = 6.7) at baseline (measured with the LPS50+ by using the normalization of the age category of 70 to 90 years for the entire sample).

For this study, we used MRI scans and cognitive ability data obtained at baseline, 1‐year follow‐up, and 7‐year follow‐up. At the 7‐year‐follow‐up the dataset still comprised 52% (*N* = 118) of the baseline sample. With these subjects, we performed our identification procedure. This subsample comprised *n* = 71 males and *n* = 47 females (mean age ± standard deviation of age; baseline: men = 70.1 ± 4.0, women = 69.7 ± 3.8; 7‐year follow‐up: men = 76.9 ± 4.0, women = 76.6 ± 3.8; mean MMSE ± standard deviation; baseline: men = 28.9 ± 1, women = 29.1 ± 0.8; 7‐year follow‐up: men = 28.1 ± 1.4, women = 28.4 ± 1.8). The IQ of the subsample was slightly higher than for the total sample at baseline (tp1: men = 134 ± 19.9; women = 131.5 ± 16.4; tp6: men = 133 ± 24.4, women = 131.5 ± 18.6).

### 2.2 Image acquisition

MRI was carried out at the University Hospital of Zurich on a 3.0T Philips Ingenia scanner (Philips Medical Systems, Best, Netherlands). All images were acquired on the same scanner using the same scanning parameters for all subjects at all time points. T1‐weighted images were recorded with a gradient echo sequence (3D turbo field echo, 160 sagittal slices, slice thickness = 1 mm, in‐plane resolution = 1 × 1 mm, FOV = 240 × 240 mm, repetition time = 8.18 ms, echo time = 3.80 ms, flip angle = 8).

### 2.3 Image processing

The longitudinal pipeline of FreeSurfer (v. 6.0, Fischl, 2012) as implemented in the FreeSurfer BIDS‐App (Gorgolewski et al., 2017) was used to obtain thickness and area measurements of cortical brain regions and volumetric measurements of subcortical structures using the Desikan‐Killiany parcellation scheme (Desikan et al., 2006). In the main analysis, we used the mean of the left and the right hemisphere for the brain measures of interest (see below). As part of our data processing pipeline, the structural MR images were visually inspected for good SNR and obvious artefacts (such as motion).

### 2.4 Preprocessing of anatomical data

For this paper, we used mainly the same anatomical measures as used in our previous age prediction paper (Valizadeh et al., 2018) applying the FreeSurfer tool (version 6.0). In this study, we estimated the following brain measures: (1) total intracranial volume (ICV), (2) total cortical thickness (CT), (3) total cortical surface area (CA), (4) cortical grey matter (CoGM), (5) cerebral white matter (CeWM), (6) cerebellar cortex (CBGM), (7) cerebellar white matter (CBWM), subcortical volumes (8) thalamus, (9) putamen, (10) pallidum, (11) caudatus, (12) hippocampus, (13) amygdala, (14) accumbens and (15) brain stem volume (midbrain, pons, medulla oblongata and superior cerebellar peduncle) using the aparc.a2009s parcellation scheme (Destrieux et al., 2010). Since the ICV measure is stable across the 7‐year period (see also below the results with respect to the RBIA analysis) and we are more interested in the individual pattern of brain tissue measures, we excluded ICV from our analyses.

### 2.5 Statistical methods used for subject identification

In this study, we have used two identification methods: (1) Linear Discriminant Analysis (LDA) and (2) Random Forest (RF). LDA is one of the simplest and most robust classification/identification techniques currently available. LDA classifiers aim at finding the best linear combination of predictors in order to optimize the separation between multiple classes. Often the primary goal of an LDA is to project a feature space onto a smaller subspace while maintaining the class‐discriminatory information. The RF algorithm has been developed by Breiman (2001) for classification demands in the context of big data analyses. The core concept of this method is to combine many decision trees on different sample sets. The final result is calculated based on the aggregation of each decision tree. This approach solves three main problems of the decision tree: (1) overfitting, (2) low observation rate compared to large feature sets and (3) handling of missing values (Biau & Scornet, 2016). In reality, RF divides the feature domain into subspace, which data could be separated by a linear model. Both algorithms, LDA and RF, provide the opportunity to estimate the relative importance of each measure (here neuroanatomical measure) for identifying subjects. The entire code has been written in R using appropriate R toolboxes (the code is available at github).

### 2.6 Training and application of the techniques

The basic idea is to train the LDA and RF algorithm for subject identification based on the data from baseline and 1‐year follow up. The training results are then used to predict individual subjects based on the same anatomical measures obtained 6 to 7 years later (at 7‐year follow‐up). Therefore, each subject is assumed to be a class with three samples. Baseline and 1‐year follow up scans are selected for training, while 7‐year follow‐up scans were used for testing. Each class (subject) was evaluated separately. The results of person identifications are reported as accuracy (Acc), sensitivity (Sense), specificity (Spec) and F1‐scores. Sensitivity (the true positive rate) reflects the proportion of correctly identified subjects. Specificity on the other hand (the true negative rate) indicates the proportion of negatives that are correctly identified (i.e., here, the number of correctly classified cases not belonging to a particular subject). Accuracy represents the proportion of truly classified subjects. The F1‐score is the harmonic mean of sensitivity (also called recall) and precision (the proportion of truly classified subjects over subjects' number in the class, also called positive predictive value). For comparison between the identification results obtained by the LDA and RF techniques, we applied a McNemar test. To identify those anatomical measures contributing most to the identification results, we calculated a stepwise LDA, and the relative importance based on the RF results.

### 2.7 RBIA

In the first step, we used ICV volume (in ccm^3^ with no decimal places) measured at baseline and compared it with the ICV volume measured at 7‐year follow‐up. As expected, there was no change in ICV during the course of 7 years and there was no overlap of the ICV values between the different subjects. Thus, a fine‐grade ICV measurement (here in ccm^3^ with no decimal places) is an ideal measure for identifying individual subjects and thus can be deemed as an ideal individual anatomical marker. In addition, this ICV stability is excellent proof for the reliability of our MRI measurements separated by 7 years. In addition, it is an excellent validation for the Freesurfer tool and our analysis pipeline.

In a second analysis step, we applied if‐then rules using the 14 brain tissue measures (1) total cortical thickness (CT), (2) total cortical surface area (CA), (3) cortical grey matter (CoGM), (4) cerebral white matter (CeWM), (5) cerebellar cortex (CBGM), (6) cerebellar white matter (CBWM), (7) thalamus, (8) putamen, (9) pallidum, (10) caudatus, (11) hippocampus, (12) amygdala, (13) accumbens and (14) brain stem volume.

We combined these 14 anatomical brain measures from the baseline measurement with if‐then rules and checked whether these rules are still valid at tp6, so that individual persons can be identified. Using these if‐then rules, we then examined whether each person has a typical or individual pattern of anatomical measures. We considered that in 7 years the anatomical measures shrink by 1%–7% (Sele et al., 2020). A verbally formulated example of a used rule with three neuroanatomical features is as follows:

If and only if CT of subject 1 at tp6 is almost identical (with a maximum deviation of 1%) of CT of subject 1 at tp1 AND CA of subject 1 at tp6 is almost identical (with a maximum deviation of 1%) of CA of subject 1 at tp1 AND CoGM of subject 1 at tp6 is almost identical (with a maximum deviation of 1%) of CoGM of subject 1 at tp1, then this combination is typical for subject 1.

To detect specific combinations of anatomical measures, which are specific for an individual subject, we sequentially increased the number of anatomical measures from 2 to 14 and examined whether we could identify individual subjects. For this, we designed four nested for‐loops to check the identification results. For obtaining a general overview, we examined all combinations of features. For example, to examine all possible combinations of two anatomical features, we examined 14!/(2![14–2]!) different combinations (total number of possible combinations of two anatomical features out of 14 = 91). With three features, we obtain 364 combinations, with four features 1001 combinations and with five features 2002 combinations. These combinations are calculated in R according to a four‐nested for‐loop (see **pseudocode 1**). It should be kept in mind that this RBIA is relatively simple but demands many computational resources. Calculation of all possible combinations of features out of 14 brain tissue measures takes about two entire days on an iMAC (4‐GHz Quad core i7, 32GB 1600‐MHz DDR3 Ram, SSD). However, the computation time for a three‐feature, five‐feature, seven‐feature or nine‐feature set lasts about 80, 659, 1463 or 1106 s.

**Pseudocode 1**: Pseudocode for calculating the RBIA. The nested for‐loops for calculating all possible combinations of anatomical features were used for comparing combinations of anatomical measures at tp1 with combinations at tp6. This formula is written in pseudocode to explain the four‐nested loops. nROI: number of ROIs (anatomical features) included in the feature set; S1:S118: loop from subject 1 to subject 118; feature. comb: is the feature set comprising the included ROIs; lower.range and upper.range: added variance due to known shrinkage and partly observed brain tissue increase during 7 years.

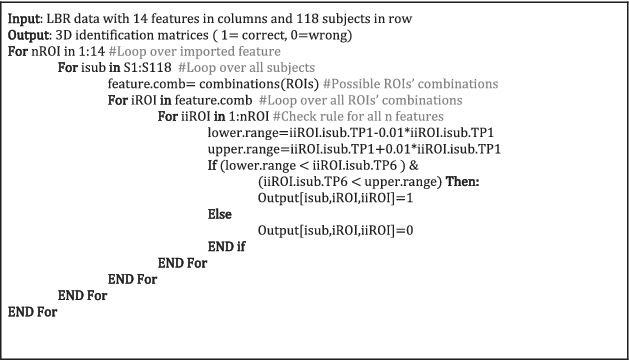

## 3 RESULTS

### 3.1 Subject identification using LDA and random Forest (RF)

Table 1 summarizes the identification results obtained from the LDA and RF approach. As one can see from this table, the identification results are perfect for LDA and excellent for RF. For RF, we have calculated two variants, one with 500 and one with 5000 trees, because it is assumed that the RF results would improve with increasing tree numbers. LDA turned out to provide perfect identification results, while RF is excellent. Applying McNemar tests to compare the accuracy obtained with LDA and RF revealed that LDA turned out to be superior. In Table 2, the results of the stepwise LDAs and RFs are shown. After 10 steps, the LDA revealed perfect identification results. In both analyses, the ranking is considerably different.

**TABLE 1:** Summary of the identification results broken down for LDA and RF

	Accuracy	Sensitivity	Specificity	F1
LDA	1.00	1.00	1.00	1.00
RF (500)	.78	.80	.99	.73
RF (5000)	.76	1.00	.76	.71

*Note*: Acc, accuracy; F1, F1‐score; Sens, sensitivity; Spec, specificity. For RF, two variants have been applied: one using 500 and one using 5000 trees for training.

Abbreviations: LDA, linear discriminant analysis; RF, random forest.

**TABLE 2:** Results of the stepwise LDA and the RF with feature ranking

	LDA feature selection	LDA classification				RF feature ranked	RF classification^a^			
		ACC	SPC	SEN	F1		ACC	SPC	SEN	F1
**1**	Hip	.01	.71	.01	.00	CeWM	.08	.92	.08	.05
**2**	Tha	.08	.92	.08	.04	Amy	.19	.97	.19	.15
**3**	CA	.24	.97	.24	.18	CT	.32	.98	.32	.26
**4**	Amy	.50	.99	.50	.43	CA	.43	.99	.43	.36
**5**	Put^b^	.81	1.00	.81	.77	CBGM^b^	.58	.99	.58	.52
**6**	CoGM^b^	.84	1.00	.84	.80	BST^b^	.66	1.00	.66	.60
**7**	BST^b^	.92	1.00	.92	.90	Pal^b^	.72	1.00	.72	.66
**8**	CBWM^b^	.98	1.00	.98	.98	Cau^b^	.78	1.00	.78	.73
**9**	Acc^b^	.99	1.00	.99	.99	Hip^b^	.73	1.00	.73	.67
**10**	Cau^b^	1.00	1.00	1.00	1.00	CBWM^b^	.74	1.00	.74	.68
**11**	CeWM^b^	1.00	1.00	1.00	1.00	Put^b^	.79	1.00	.79	.74
**12**	Pal^b^	1.00	1.00	1.00	1.00	CoGM^b^	.76	1.00	.76	.72
**13**	CBGM^b^	1.00	1.00	1.00	1.00	Acc^b^	.79	1.00	.79	.74
**14**	CT^b^	1.00	1.00	1.00	1.00	Tha^b^	.76	1.00	.76	.71

*Note*: The anatomical features are listed from top to bottom according to their importance for subject identification.

Abbreviations: LDA, linear discriminant analysis; RF, random forest.

^a^ 500 trees are used for training.

^b^ Significant difference in Mcnemar test.

### 3.2 Subject identification using the RBIA

Table 3 shows the number of possible combinations of neuroanatomical features out of the used 14 brain tissue measures separately for each feature set (from 2 to 14 features). This table also shows the number (and percent) of those features revealing perfect subject identifications. These percent values represent the average of correct identification across the ‘number of possible identifications’ separately for each feature set. Thus, for the two‐feature set, there are 91 possible combinations, 10,738 (91 * 118) possible identifications, and 4297 correctly identified subjects, which on average amounts to 40% correct identifications. For the five‐feature sets, there were 2002 different feature sets with five anatomical features. This amounts to 236,236 possible identifications (2002 * 118). Of these 236,236 possible identifications, 224,937 combinations revealed perfect subject identification, amounting to 95% overall correct subject identification. Increasing the number of included features to 10 results in 1001 possible 10‐feature combinations reveals a total of nearly 100% correct subject identifications (99.99%). Only 535 of all possible 118,118 combinations demonstrated wrong identification. Absolutely perfect identification (with no wrong identifications) is achieved with all 14 features (see also Table 3).

**TABLE 3:** Number of possible combinations comprising 2–14 features (*n* of possible combinations) for the ‘rule‐based‐identification‐approach (RBIA)’

Number of features	*n* of possible feature combinations	*n* of possible identifications	*n* of correctly identified combinations	*n* of wrongly identified combinations	Proportion of correctly identified subjects
2	91	10,738	4297	6441	.40
3	364	42,952	34,683	8269	.81
4	1001	118,118	107,928	10,190	.91
5	2002	236,236	224,937	11,299	.95
6	3003	354,354	344,256	10,098	.97
7	3432	404,976	397,848	7128	.98
8	3003	354,354	350,406	3948	.99
9	2002	236,236	234,550	1686	.99
10	1001	118,118	117,583	535	1.00
11	364	42,952	42,834	118	1.00
12	91	10,738	10,722	16	1.00
13	14	1652	1651	1	1.00
14	1	118	118	0	1

*Note*: Shown are also the ‘number of possible identifications’ (*n* of possible feature combinations * 118), the ‘number of correctly identified combinations’, the ‘*n* of wrongly identified combinations’, and the ‘proportion of correctly identified subjects’.

**FIGURE 1:**
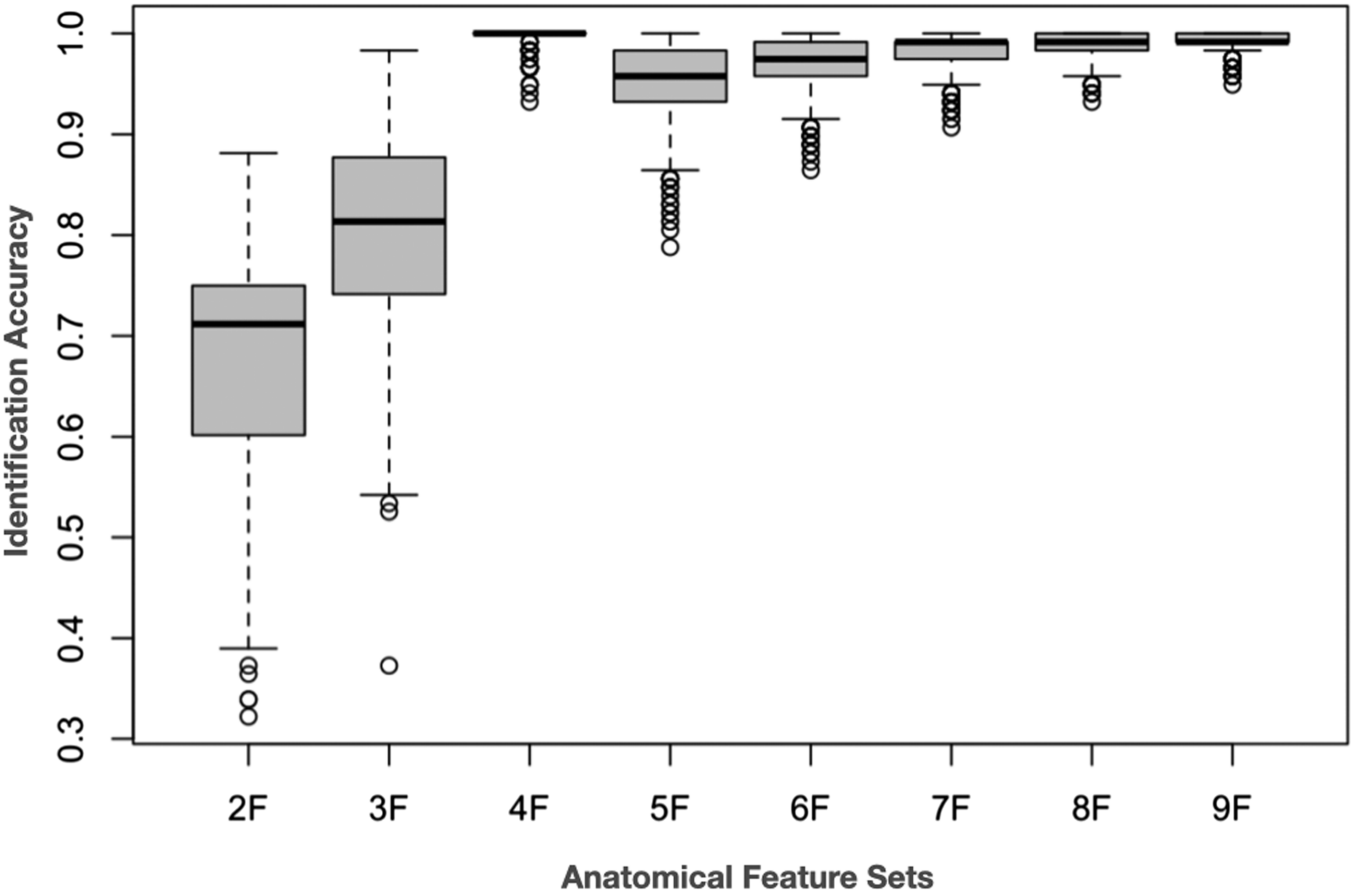
Distribution of accuracy separately for each group of feature sets for the ‘rule‐based‐identification approach’ (RBIA). On the abscissa, the different feature sets are shown (2F = two‐feature set, 9F = nine‐feature set). Shown are box plots with the maximum and minimum of accuracies. There is a 100% correct identification starting from the four‐feature set on.

Table 4 shows the two best and the two worst identification rates broken down for each feature set. From the 91 possible two‐feature combinations two feature sets revealed good identification results (CoGM ∧ Acc: 88%, Pal ∧ Acc: 87%). The best three‐feature sets reveal excellent subject identification accuracy (CeWM ∧ Cau ∧ Acc: 98%, CoGM ∧ Pa ∧, Acc: 97%). For the four‐feature sets, we obtained in total five feature sets yielding perfect subject identification. For the five‐ to nine‐feature sets we obtained an increasing number of feature sets with 100% correct person identification. In Table 4 we have arbitrarily chosen two feature sets with perfect person identification for the four‐ to nine‐feature sets. Figure 1 shows the distribution of accuracies for all feature sets.

**TABLE 4:** Shown are the best and worst combinations with respect to the accuracy (ACC) of subject identification broken down for each feature‐set 2–9 in the context of the ‘rule‐based‐identification‐approach (RBIA)’

Number of features	Rank	Best		Worst	
		Combinations of anatomical features	ACC	Combinations of anatomical features	ACC
2	1	CoGM, Acc	88%	CT, Tha	32%
	2	Pal, Acc	87%	CT, CoGM	34%
3	1	CeWM, Cau, Acc	98%	CT, CA, CoGM	37%
	2	CoGM, Pal, Acc	97%	CA, CBGM, BST	53%
4^1^	1	CT, CA, CoGM, CeWM	100%	CT, CA, CoGM, CBGM	93%
	2	CT, CA, CoGM, Acc	100%	CT, CA, CoGM, Cau	94%
5^2^	1	CT, CA, CeWM, Cau, Acc	100%	CT, CA, CoGM, CBWM, BST	79%
	2	CT, CA, CBGM, Tha, Acc	100%	CT, CA, CoGM, CBGM, BST	81%
6^3^	1	CT, CA, CoGM, CeWM, Cau, Acc	100%	CT, CA, CoGM, CBGM, CBWM, BST	87%
	2	CT, CA, CoGM, CBGM, Tha, Acc	100%	CT, CA, CoGM, CBGM, Cau, BST	87%
7^4^	1	CT, CA,CoGM, CeWM, CBGM, Tha, Acc	100%	CT, CA, CoGM, CBGM, Tha, Cau, BST	90%
	2	CT, CA, CoGM, CeWM, CBGM, Put, Acc	100%	CT, CA, CoGM, CBGM, Put, Cau, BST	92%
8^5^	1	CT, CA, CoGM, CeWM, CBGM, CBWM, Tha, Acc	100%	CT, CA,CoGM, CBGM, Tha, Put, Cau,BST	93%
	2	CT, CA, CoGM, CeWM, CBGM, CBWM, Put, Acc	100%	CT, CA, CoGM,CeWM, CBWM,Tha,Put, BST	94%
9^6^	1	CT, CA, CoGM, CeWM, CBGM, CBWM, Tha, Put, Acc	100%	CT, CA, CoGM, CeWM, CBGM, Tha, Put, Cau, BST	95%
	2	CT, CA, CoGM, CeWM, CBGM, CBWM, Tha, Pal, Hip	100%	CT,CA,CoGM,CeWM, CBWM, Tha, Put, Cau, BST	96%

*Note*: From feature sets with 10 features, the accuracy is perfect for all possible 10‐feature sets. (1) Number of four‐feature combinations with 100% accuracy: 5; (2) number of five‐feature combinations with 100% accuracy: 67; (3) number of six‐feature combinations with 100% accuracy: 284; (4) number of seven‐feature combinations with 100% accuracy: 5649; (5) number of eight‐feature combinations with 100% accuracy: 934 and (6) number of nine‐feature combinations with 100% accuracy: 898.

## 4 DISCUSSION

In this study, we investigated whether it is possible to identify individual subjects based on neuroanatomical characteristics measured 7 years previously. For this purpose, we applied three different methods for subject identification. Two methods are standard classical machine learning techniques with different strengths, weaknesses, and mathematical requirements (linear discriminant analysis: LDA and random forest: RF). As a third approach, we have developed a new method (RBIA) that combines the neuroanatomical characteristics by using if‐then rules to evaluate whether these logical combinations can be used to identify individuals based on neuroanatomical characteristics from MRI images that were acquired 7 years earlier.

LDA produced perfect identification results, while RF achieved very good results. Both algorithms showed that approximately 10 brain tissue measures are necessary to achieve excellent identification results. The stepwise analyses revealed that the cerebral WM, cortical thickness, cortical area and several subcortical measures strongly contribute to subject identification.

The newly developed RBIA demonstrates, in our view, the most interesting results. Generating all possible two‐feature sets, which amounts to 10,738 sets, produced on average 40% perfect subject identifications. The best two two‐feature sets achieved 88% and 87% correct subject identification. The four‐feature sets (in total 118,118 sets) produced on average 91% correct subject identification. Five four‐feature sets showed perfect subject identification. Increasing the number of features improved subject identification substantially. With 10 features, nearly perfect subject identification was achieved. Using all 14 features, we obtained perfect subject identification.

It should also be mentioned that, as expected, the cranial volume (ICV) remained stable over the 7‐year period and so can also be used as a feature to achieve perfect person identification. Besides the fact that this morphological feature is (as expected) stable over the course of 7 years, this finding emphasizes that our longitudinal MRI measurement, the FreeSurfer parcellation and our analyses pipeline are reliable.

To that extent, this study again shows that each subject has an individual combination of neuroanatomical characteristics or, in other words, that each brain is unique in terms of morphological characteristics (Valizadeh et al., 2018; Wachinger et al., 2014, 2015). It is remarkable in this context that even 7‐year‐old neuroanatomical characteristics can be used for subject identification. The individuality of the brain tissue measures is due to genetic, non‐genetic and experience‐related influences. Besides the for us most important finding that the human brain is highly individual in terms of neuroanatomical features, brain measures might be used in the future to identify an individual subject. Thus, these neuroanatomical measures have the potential to complement techniques currently used for subject identification (e.g., fingerprints and eye features). The quality of individual identification based on neuroanatomical features is at least as good as the classic fingerprint analysis. Potentially, neuroanatomical characteristics could be used in forensics, for example, to verify the identity of unknown corpses. Although it is known that postmortem brains shrink a bit due to water loss but then increase their brain weight after formalin fixation by about 1.5% (Witelson et al., 2006), postmortem brains can be well measured with MRI, which produces similar results to more direct measurement methods (Peters et al., 2000). However, we would like to emphasize that we are more interested in the obvious ‘individuality’ of the human brain.

Some remarks regarding the limitations and methodological issues are necessary. A caveat regarding LDA in the context of our data corpus is that this algorithm projects the data into a low‐dimensional space and minimizes the intra‐class variance while maximizing the inter‐class difference. However, by nature, the linear model has limited accuracy and discrimination when dealing with high‐dimensional data, as in our case. A further problem is that the intra‐class variance is very small (here, the variance within each subject) and therefore excellent discrimination between subjects is highly likely. RF is based on logical rules, which are sequentially combined. Thus, it needs no strong mathematical prerequisites and works well with high‐dimensional data. The best approach, in our view, is the rule‐based approach, which we have developed for this study. This approach is relatively simple; however, it requires a lot of computing resources. The advantage of this approach is that the mathematical prerequisites for application are relatively sparse. This is particularly important since the anatomical features are differently related to each other. From previous studies, we know that several brain components (e.g., cerebral WM and the accumbens volume) are scaled out of proportion while others are scaled less than proportional with brain volume (Jäncke et al., 2019). Therefore, a linear combination of such anatomical features as used by LDA might induce prediction errors. A further limitation of our study is that we entirely relied on the brains of older subjects. Whether the subject identification works similarly well in the brains of younger individuals remains to be investigated. Above all, it is not yet clear how much true longitudinal change of the neuroanatomical measures or their relationships can be expected in the brains of younger adults over 7 years. Hence, it will be interesting for future studies to verify whether these relations are preserved in adolescents and children and are stable into adulthood.

## 5 CONCLUSION

Applying two standard classification algorithms (linear discriminant analysis: LDA and random forest: RF) to identify individual subjects based on 7‐year‐old neuroanatomical feature measurements produced excellent (for LDA) and very good (for RF) subject identification results when using at least 10 anatomical features. Applying a newly developed ruled‐based identification algorithm (RBIA), the subject identification became perfect even with a subset of only four features. The identification results substantially improved by increasing the number of included neuroanatomical features. With 14 features, we achieved perfect subject identification. This study shows again that the human brain is highly individual in terms of neuroanatomical features and provides a reliable and valid option for brain fingerprinting.

## CONFLICT OF INTEREST

The authors have no conflict of interest to declare.

## AUTHOR CONTRIBUTIONS

Lutz Jäncke: supervision of data acquisition, experimental design, data analysis and writing of the manuscript; statistical analysis; Seyed Valizadeh: supervision of data acquisition, data analysis and reviewing of the manuscript; coding of statistical analysis.

### PEER REVIEW

The peer review history for this article is available at https://publons.com/publon/10.1111/ejn.15770.

## Data Availability

The R analysis code and the associated data tables will be made available by the authors. The raw MR image data underlying this article are not publicly available and can only be accessed via collaborations with the URPP Dynamics of Healthy Aging.
